# Sodium – a systematic review for Nordic Nutrition Recommendations 2023

**DOI:** 10.29219/fnr.v68.10319

**Published:** 2024-01-31

**Authors:** Antti Jula

**Affiliations:** Department of Clinical Medicine, University of Turku, Turku, Finland

**Keywords:** sodium, sodium chloride, salt, blood pressure, hypertension, nutrition recommendations

## Abstract

Blood pressure (BP) rises along with increasing sodium intake from early childhood to late adulthood, and leads to hypertension among most men and women living in Nordic and Baltic countries. Elevated BP is the leading global risk factor for premature deaths and disability-adjusted life-years. A reduction in sodium intake is essential in the prevention of hypertension in individuals, in the lowering of BP levels, in the treatment of hypertensive individuals, and in decreasing risks associated with elevated BP. There is a progressive linear dose-response relationship between sodium intake and BP beginning from a sodium intake of less than 0.8 g/day. Sodium reduction decreases BP linearly by a dose-response manner down to a sodium intake level of less than 2 g/day. Randomised intervention studies with a duration of at least 4 weeks confirm the efficiency and safety of reducing blood sodium intake to a level of less than 2 g/day. Results from prospective cohort studies show that higher sodium intake is positively associated with an increased risk of stroke and cardiovascular events and mortality among the general adult population, and the associations are linear in studies using proper sodium assessment methods. Analyses assessing sodium intake using at least two 24-h urine samples have shown a linear positive relationship between sodium intake and the risk of a cardiovascular event or death. Based on an overall evaluation of the available data, a limitation of the sodium intake to 2.0 g/day is suggested for adults. The optimal sodium intake level would be probably about 1.5 g/day. Sodium intake recommended for children can be extrapolated from the recommended sodium intake for adults. According to national dietary surveys, the average sodium intakes in Nordic countries range in adult men from 3.6 to 4.4 g/day and in adult women from 2.6. to 3.2 g/day, and in Baltic countries in men from 2.6 to 5.1 g/day and in women from 1.8 to 3.6 g/day.

## Popular scientific summary

- Sodium plays an important role for the concentration of the fluid outside the cells in the body and for electrical signalling of the nerves, muscles, and heart.
- The major source of sodium in the diet is salt added to processed foods, including bread, meat products, cheese, and ready meals.
- A lack of sodium, hyponatraemia, is seldom caused by dietary restriction.
- Intake of sodium increases blood pressure in a dose-dependent manner.
- High intake of sodium is associated with increased risk of cardiovascular disease and stroke.

Sodium chloride (NaCl; table salt), the dominant and most important source of dietary sodium, is used as a food ingredient or condiment. Sodium is also found in unprocessed foods, but usually in very low concentrations. One gram of salt corresponds to about 0.4 g sodium, and 1 g sodium is equivalent to 2.5 g salt. One millimole of sodium corresponds to 23 mg and is equivalent to about 58 mg sodium chloride. The aim of this scoping review is to describe the totality of evidence for the role of sodium for health-related outcomes as a basis for updating dietary reference values (DRVs) for the Nordic Nutrition Recommendations 2023 (NNR 2023) (Box 1).

*Box 1.* Background papers for Nordic Nutrition Recommendations 2023

- This paper is one of many scoping reviews commissioned as part of the Nordic Nutrition Recommendations 2023 (NNR2023) project (Blomhoff et al, NNR report)
- The papers are included in the extended NNR2023 report but, for transparency, these scoping reviews are also published in Food & Nutrition Research
- The scoping reviews have been peer reviewed by independent experts in the research field according to the standard procedures of the journal
- The scoping reviews have also been subjected to public consultations (see report to be published by the NNR2023 project)
- The NNR2023 committee has served as the editorial board
- While these papers are a main fundament, the NNR2023 committee has the sole responsibility for setting dietary reference values in the NNR2023 project

## Methods

This scoping review on sodium as salt follows the protocol developed within the NNR2023 project (1). The sources of evidence used in the chapter follow the eligibility criteria described previously (2). Seven qualified systematic reviews (SRs) on sodium were found (3). These include the recent scientific background to reference values for the USA (children 2015 and adults and children 2018), Australia and New Zealand (2017), European Food Safety Authority (EFSA 2019), and the systematic review by World Cancer Research Fund (WCRF) on preservation and processing (2018).

The search was conducted on 19.09.2021 on PubMed, and contained the following terms sodium [Title] AND (“2011”[Date - Publication]: “3000”[Date - Publication]) AND review [Publication Type] AND Humans[Filter] AND (“Diet” OR “Dietary” OR “Food” OR “Nutrition” OR “Nutritional”). The primary search was complemented by a new search on 31.1.2022.

## Physiology

Sodium ion (Na^+^) is the major cation in the extracellular fluid (ECF). Together with the chloride anion (Cl^-^), they are the main contributors of the ECF osmolality (4). The ECF volume and the equilibrium between intracellular and extracellular osmolality is controlled by systems transporting sodium into the cell and by the energy-dependent sodium pump (Na^+^/K^+^-ATPase) that pumps sodium out of the cell in exchange for potassium. The differences in transmembrane distribution and activity gradients of sodium and potassium create a polarised cell membrane potential. Ion channels located in the cell membranes open in response to stimuli and allow ion flow and depolarisation of the cell membrane. The ion flow across the membranes is fundamental for the entry of water, glucose and amino acids into the cell, and for the electrical signalling of the nervous system, muscles, and heart (4).

The estimated body pool of sodium of an adult human is 1.3–1.5 g/kg of body weight (4), and half of it is located in ECF (2). Of the body total sodium, approximately 10.0% is in plasma, 30.0% in the interstitial fluid, 2.5% within cells, 2.5% in transcellular spaces, and 55.0% is bound in connective tissue, cartilage, bone, and soft tissues (5, 6). Approximately 98% of the ingested sodium is absorbed (7) and, on average, 93% of the ingested sodium is excreted in urine (8). Small amounts of sodium are lost in the faeces representing less than 2% of sodium intake (4). Dermal losses are influenced by sodium intake, sweat rate, hydration status and degree of heat acclimation, and represent the rest of the sodium losses (4). During profound sweating or in massive diarrhoea or vomiting, the extrarenal loss of sodium may become clinically important.

Renal sodium excretion is the difference between sodium filtered by the glomeruli and its total tubular reabsorption (5). Normally 98–99.99% of the filtered sodium is reabsorbed. The major mediators of sodium excretion are arterial blood pressure (BP) together with several neurohumoral factors. At the tubular level angiotensin II, aldosterone, norepinephrine and insulin stimulate sodium reabsorption. Dopamine, cAMP, cardiac natriuretic peptides, kinins, nitric oxide and some prostaglandins increase sodium excretion.

## Assessment of nutrient status

There are no sensitive and specific biomarkers for estimation of sodium status of the body. The osmoregulatory systems control water intake and excretion, and keep the plasma sodium concentration in its normal range (135–145 mmol/L). Hyponatraemia may be caused by water dilution in the syndrome of inappropriate antidiuretic hormone excretion (SIADH) and is induced by some drugs and diseases, or when drinking large amounts of water in a short time (more than 1 l/h over several hours), by sodium and water retention in severe heart failure, and by true sodium depletion. Dietary causes for hyponatraemia are rare.

Sodium intake can be estimated by means of dietary questionnaires (24-h dietary recalls, food diaries or food frequency questionnaires [FFQ]) or by measuring 24-h urinary sodium excretion. There is a poor agreement with low correlations between sodium intake estimates based on dietary questionnaires and the gold standard 24-h urinary sodium excretion (9–11). Dietary questionnaires may underestimate sodium intake (9–11). In a pooled analyses of five large validation studies, average underreporting of sodium intake was 28–39% for FFQ and 4–13% for dietary recalls. The FFQs were not specific for sodium intake, and they had limited ability to assess discretionary salt intake (9–11). At the individual level, higher 24-h urinary sodium excretion and higher body mass index (BMI) are strongly associated with underreporting of sodium intake, probably mostly as a consequence of underreporting of energy intake (9, 11).

Sodium intake varies day-by-day. Even at a constant intake the daily variation in sodium excretion is high, and, sodium excretion shows also an aldosterone-dependent weekly rhythm (12–14). At least three but up to seven complete 24-h urine collections are needed to accurately estimate an individual’s short-term sodium intake (12, 13). Multiple 24-h urine collections over several years are needed to reflect an individual’s long-term sodium intake (7, 12). Complete collections of 24-h urinary sodium representing days of usual sodium intake (including weekend and week days) and possible seasonal variations is the gold standard to estimate average population sodium intake (7, 12).

Estimation of daily sodium intake from spot urine by using formulas based on age, urinary sodium, urinary creatinine, weight and age (Kawasaki), plus height (Tanaka), plus urinary potassium, and BMI instead of weight and height (Intersalt), has been used to assess cardiovascular risk in association with sodium intake (15–17). Correlations between daily sodium intake estimates from spot urine and 24-h urinary sodium are low and exhibit various sources of biases (7, 12). According to an analysis based on the Trials of Hypertension Prevention (TOHP) follow-up study, all three formulas overestimate at lower levels and underestimate at higher levels of sodium intake (17). The Kawasaki and Tanaka formulas overestimated on an average by 1,299 and 107 mg/day, respectively, and the Intersalt formula underestimated by 78 mg/day the average measured 24-h urinary sodium excretion over the trial period (17). The estimates of sodium intake by the three formulas were done by using sodium, creatinine, and potassium (Intersalt) concentrations measured from the 24-h urine. The results of the study suggest that sodium-independent variables in the formulas contribute to the biased estimates of sodium intake (17). One of them is urinary creatinine, which increases along with higher lean body mass (muscle mass), higher dietary protein intake, and higher levels of physical activity (18). BP level, secondary forms of hypertension, heart failure, medication and variation in the daily rhythm of sodium intake modify diurnal sodium excretion. This may further lead to serious systematic biases in the estimation of an individual’s sodium intake from spot-urine and, consequently, in the estimation of relations between sodium intake and cardiovascular disease (CVD). The J- or U-shaped relationships between sodium intake estimates and CVD disappear, and are linear, when repeated measurements of 24-h urinary sodium excretion is used in the assessment of sodium intake (17). Sodium intake estimated from spot-urine is not a valid method to assess sodium intake at the individual level, and cannot be used in the assessment of BP and cardiovascular risk in relation to sodium intake or in the comparisons of sodium intake across and within populations. (4, 7, 12, 19).

## Dietary intake in the Nordic and Baltic countries

Approximately 60 to 70% of sodium in the diet comes from salt added to processed foods such as bread, bakery and other cereal products, meat and fish products, cheese, and ready meals such as pizza, pie, and soups (20–22). The contribution of sodium from added salt in cooking and at the table varies, but on average this intake constitutes approximately 10 to 20% of the total salt intake (20, 21).

Data on the dietary intake of sodium in Nordic and Baltic countries are based on the latest national dietary surveys done between the years 2007 and 2019, with 1,011 to 3,916 adult participants per survey, and with a participation rate ranging from 33 to 90%. Dietary data was collected by food records in Denmark (7-day) and Sweden (4-day), and with two separate 24-h dietary recalls in other countries (23). Daily mean sodium intakes were 4.4 g/day (11.0 g salt) in men and 3.2 g/day (8.0 g salt) in women in Denmark, 3.8 g in men (9.5 g salt) and 2.8 g (6.9 g salt) in women in Finland, 3.6 g/day (9 g salt) in men and 2.5 g/day (6.25 g salt) in women in Norway, 3.8 g (9.4 g salt) in men and 2.6 g (6.5 g salt) in women in Iceland, 3.6 g (9.0 g salt) in men and 2.7 g (6.9 g salt) in women in Sweden, 2.6 g (6.5 g salt) in men and 1.8 g (4.5 g salt) in women in Estonia, 3.2 g (7.9 g salt) in men and 2.4 g (6.0 g salt) in women in Lithuania, and 3.3 g (8.3 g salt) in men and 2.5 g (6.3 g salt) in women in Latvia (22, 23).

The contribution from discretionary salt intake – such as that from extra salt added to meals, etc. – is generally not included in these estimates. Data from Finnish population studies suggest that sodium intake assessed from 24 h and 48 h dietary recalls give similar estimates of the mean level of sodium intake as determinations of 24 h urinary sodium excretion (24). Salt intake was estimated based on food balance sheets, and the use of salt at the table was recorded (25). According to the latest Latvian dietary survey, 24-h urinary sodium excretions of 541 women and 470 men aged 19–64 years were 41 to 54% higher than daily sodium intake estimates made by means of dietary recalls, and were 5.1 g/day (12.8 salt) in men and 3.6 g/day (8.9 g salt) in women (25). Therefore, dietary estimates of population sodium intake should always be validated against 24-h urinary sodium excretion.

## Health outcomes relevant to Nordic and Baltic countries

### Deficiencies

Sodium deficiency does not normally exist in healthy individuals. Acute deficiency, hyponatraemia, can be caused by extreme and prolonged sweating in combination with large fluid intakes devoid of sodium or by massive vomiting and chronic diarrhoea. Severe adrenal insufficiency, excessive use of diuretics, sodium-losing kidney disease, and diabetic ketoacidosis may lead to sodium and water depletion. Clinical symptoms include: muscle seizures, loss of appetite, and circulation disturbances. Severe deficiency can result in coma and death.

### Toxicities

Acute toxicity with fatal outcomes has been reported with single doses ranging from 6.8–10.2 g up to 400 g of sodium in adults and from 5 to 7 g in small children (26). The fatal outcomes were associated with severe hypernatremia and with plasma sodium levels ranging from 151 mmol/L to 255 mmol/L. A much smaller amount of an acute sodium load could be detrimental for subjects with heart failure, renal failure or decompensated liver cirrhosis. European Food Safety Authority defines hypernatremia as serum sodium concentration >145 mmol/L, and describes similar symptoms as hyponatraemia as well as headache, confusion, fever, nausea and vomiting (4).

### Obesity and sodium

Obesity is associated with a higher sodium intake and increased sodium sensitivity (27–31). In a systematic review and meta-analyses of cross-sectional studies, a higher sodium intake was associated with overweight and obesity in adults and in children, and with abdominal obesity in adults (26). Insulin resistance, hyperinsulinemia, overactivity of the sympathetic nervous and renin-angiotensin systems are common features of obesity. Higher plasma insulin, aldosterone and cortisol levels enhance sodium tubular reabsorption and increase sodium load (28–31).

### Hypertension

Elevated systolic BP was in 2017 the leading global risk factor for 10.4 million premature deaths and 218 million disability-adjusted life-years (32). Elevated BP increases the risk of cerebrovascular disease, ischemic heart disease, heart failure, peripheral artery disease, and end-stage renal disease. The risk of cardiovascular mortality rises without any threshold from BP levels less than 115/75 mmHg. The risk of fatal stroke is three-fold, and that of fatal myocardial infarction is two-fold, for a rise of 20 mmHg in systolic blood pressure (SBP) and 10 mmHg in diastolic blood pressure (DBP) (33). In the Framingham follow-up study, the life-expectancies of 50-year-old men and women with a BP less than 120/80 mmHg were 5.1 and 4.9 years longer, respectively, as compared with men and women with SBP or DBP at least 140 mmHg and 90 mmHg (34). According the FinHealth 2017 health examination survey, the prevalence of hypertension, as determined by SBP at least 140 mmHg or DBP at least 90 mmHg or use of antihypertensive medication, increased along with age of men/women and was 28/11% in the age group of 30–39 years, 40/23% in the age group of 40–49 years, 62/47% in the age-group 50–59 years, 74/69% in the age-group of 60–69 years, 79/84% in the age-group of 70–79 years, and 80/87% in the age group of 80 years or more (35). Approximately 40% of the subjects treated for hypertension have their BP levels below 140/90 mmHg. Population-based BP data measured with exactly similar methods are difficult to find from other Nordic and Baltic countries. However, the existing data suggest that age-adjusted BP levels of men and women are quite similar in Nordic countries and somewhat higher in Baltic countries (36). Elevated BP also carries a high burden for increased cardiovascular morbidity and mortality in the Nordic and Baltic countries.

BP starts to rise in early childhood and leads most often to hypertension later in life as a consequence of functional and structural microvascular and macrovascular alterations. Hypertension is characterised by rarefication of the capillary density, increased contractility of the resistance arteries, stiffening of the large conduit arteries, and changes in the structure and function of the heart. Changes in the microvasculature lead to increased peripheral resistance. Increased fibrosis and decreased elastin to collagen ratio in the arterial wall of large conduit arteries (aorta) lead to their stiffening and to changes in the pulsatile components of the pulse wave, clinically observed by changes (peaking) in the central pulse-wave form, increased aortic pulse wave velocity, and, robustly, by increased pulse pressure (difference between SBP and DBP) and SBP. High intake of sodium plays a significant role in the long-run process of the development of hypertension (37–40). Increased intracellular calcium in vascular smooth muscle cells, induced primarily by high sodium intake and sodium retention, may be one of the key factors behind increased contractility of resistance arteries, impaired vascular endothelial function, and increased peripheral resistance in sodium-sensitive hypertension (41, 42). High sodium intake may induce stiffening of the large arteries and structural changes of the left ventricle also by pressure-independent mechanisms (41, 43).

#### Adults

Observational studies have shown that hypertension is rare and that BP does not normally rise with age in populations with a very low sodium intake (<0.8 g/day) (44). In the Intersalt study, the relationship between 24-h sodium and potassium excretion and BP was investigated. The study included 10,000 men and women aged 20–59 years from 52 centres around the world. The median urinary sodium excretion varied from 0.9 mmol/day (0.02 g/day) to 242 mmol/day (5.57 g/day) between the centres. In four centres (Brazil: Xingu and Yanomamo; Papua New Guinea; Kenya) with low sodium excretion (0.02–1.31 g/day, lowest in the two centres in Brazil and highest in Kenya), the BP levels were low and practically no hypertension was observed. Across the four centres, the average BP increased from 96.0/60.6 mmHg to 113.3/66.0 mmHg along with increasing sodium excretion. No age-related increase in BP was observed in the two centres with the lowest sodium excretion (Xingu and Yanomamo), and the age-related rise was only small in the other two centres (Papua New Guinea and Kenya). According to within-population analyses of all 52 centres, a 100 mmol (4.35 g) higher 24-h urinary sodium was associated with a 3/0 to 6/3 mmHg higher SBP/DBP (analyses with and without BMI (45). In cross-population analyses, a 100 mmol higher 24-h urinary sodium was associated with a 5–7/2–4 mmHg higher SBP/DBP (45) and with an estimated mean difference of 10–11/6 mmHg in SBP/DBP between 55 and 25 years of age. Increased BMI and heavy alcohol intake were strongly related to increased BP. A positive linear correlation between sodium intake and BP has been confirmed in other cross-sectional studies and meta-analyses of observational studies in adults as well as in children and adolescents (46–48). A positive nonlinear association was observed in a large study where daily sodium intake was estimated from spot-urine (49).

Several meta-analyses of clinical trials of dietary salt reduction have been published, and they differ in scope and inclusion criteria. The meta-analyses of Agency for Healthcare Research and Quality (AHRQ) in the United States of America (US) identified 47 comparisons that met their inclusion criteria (50). The analyses were based on parallel randomised controlled trials (RCTs) and on crossover RCTs with a washout period of at least 2 weeks. The net differences in sodium intakes between high and low sodium groups or phases ranged from 0 to 120 mmol/day (0 to 2,760 mg/day) with a weighted mean difference of 42 mmol/day (966 mg/day). BP measurements in the included studies were made in sitting or supine position (the intervention effect on supine and sitting BP may differ from each other). Several studies with hypertensive subjects using BP-lowering medication, also diuretics, were included. A decrease in BP-lowering medication was one of the primary endpoints in one study showing halving of BP-medication during sodium restriction. Yet, the net BP-changes between the sodium lowering and the usual sodium intake arms were used for the analyses. There were also errors in numerical values of the net BP changes between the sodium restriction and control groups. In the analyses of all trials with hypertensive and normotensive participants combined, sodium reduction decreased SBP by 3.2 mmHg (95% confidence interval [CI]: −4.1 to −2.4 mmHg) and DBP by 2.3 mmHg (95% CI: −2.9 to −1.6 mmHg). SBP decreased by 4.1 mmHg (95% CI: −5.2 to −3.1 mmHg) and DBP by 2.6 mmHg (95% CI: −3.3 to −1.9 mmHg) in hypertensive participants. In normotensive participants, SBP decreased by 1.5 mmHg (95% CI: −2.8 to −0.3 mmHg) but DBP did not decrease significantly (−0.6 mmHg; 95% CI: −1.3 to +0.1 mmHg). A larger difference in sodium intakes between high and low sodium groups or phases and a higher mean age was associated with a larger BP decrease.

The report of the EFSA Panel on Nutrition, Novel Foods and Food Allergens identified 32 RCTs and 35 comparisons that met their inclusion criteria (4). The analyses were based on seven parallel RCTs and 25 cross-over trials. The net differences in sodium intakes between high and low sodium groups or phases ranged from 13 to 285 mmol/day (300 to 6,560 mg/day), with a median value of 72 mmol/day (1,656 mg/day). In the analyses of all trials with hypertensive and normotensive participants combined, sodium reduction decreased SBP by 3.9 mmHg (95% CI: −5.1 to −3.1 mmHg) and DBP by 2.0 mmHg (95% CI: −2.8 to −1.2 mmHg). A larger effect was found in hypertensive than normotensive subjects. SBP decreased by 5.6 mmHg (95% CI: −8.1 to −3.1 mmHg) and DBP by 2.9 mmHg (95% CI: −4.2 to −1.6 mmHg) in hypertensive participants. In normotensive participants, SBP decreased by 2.0 mmHg (95% CI: −3.3 to −0.7 mmHg) and DBP by 0.9 mmHg 95% CI: −1.6 to −0.2 mmHg). According to the dose-response analyses of eligible trials, for each 100 mmol /day (2.3 g/day) increase in mean 24-h urinary sodium excretion, mean SBP increased by 5.3 mmHg (94% CI: 3.6 to 6.9 mmHg) and mean DBP increased by 2.6 mmHg (95% CI: 1.6–3.7 mmHg).

Graudal et al. studied effects of low versus high sodium diets on BP, renin, aldosterone, catecholamines, and serum lipids (51). The Cochrane Database systematic review included 195 intervention studies published between 1973 and 2018, 12,296 individuals, and sodium restriction durations of 3 to 1,100 days. The effects on BP were shown separately for white, black and Asian participants. A mean sodium reduction from 203 mmol/day (4,670 mg/day) to 65 mmol/day (1,490 mg/day) decreased SBP in hypertensive white participants by 5.7 mmHg (95% CI: −6.7 to −4.7 mmHg; 3,998 participants, 88 trials) and DBP by 2.9 mmHg (95% CI: −3.4 to −2.3 mmHg; 4,032 participants, 89 trials). In normotensive white participants SBP decreased by 1.1 mmHg (95% CI: −1.7 to −0.6 mmHg; 5,982 participants, 95 trials) but DBP was unchanged (+0.1 mmHg; 95% CI: −0.4 to +0.4 mmHg; 6,276 participants, 96 trials). The effects on BP were largely similar in black and Asian participants. During sodium restriction, serum cholesterol increased by 0.08 mmol/l (95% CI: 0.04 to 0.13 mmol/l; 917 participants in 27 trials), triglycerides increased by 0.13 mmol/l (95% CI: 0.05 to 0.21 mmol/l; 712 participants in 20 trials), LDL-cholesterol tended to increase (0.06 mmol/l; 95% CI: −0.03 to 0.15 mmol/l), but no significant changes were seen in HDL-cholesterol. During low sodium intake, renin (2,904 participants, 82 trials), aldosterone (2,506 participants, 66 trials) and noradrenalin (878 participants, 35 trials) were higher than during high sodium intake. A weakness of the analysis of trials on normotensives and of studies on the sodium and BP regulating hormones was their short duration (usually not more than 14 days, often 3–7 days). Most of the studies were experimental and explored sodium and BP regulating systems, sodium sensitivity and BP reactivity in healthy, young voluntary subjects during a very low sodium intake of 20–30 mmol/day (0.5–0.7 g/day) and during a high sodium intake of usually 200–300 mmol/day (4.6–6.9 g/day), but up to 800 mmol/day (18.4 g/day). The changes in renin, aldosterone and noradrenaline levels can be estimated to be physiological under these circumstances. Furthermore, included studies exploring effects of sodium intake on BP reactivity showed that BP rise to noradrenaline infusion was smaller during strict sodium restriction than after a heavy sodium load (52, 53).

The meta-analyses of Aburto et al. included randomised controlled studies that had a duration of at least 4 weeks, achieved a difference of at least 40 mmol/day (0.9 g/day) in sodium intake between intervention and control and measured 24-h urinary sodium excretion (54). They identified 36 studies with 49 comparisons, and found that a reduction in sodium intake reduced SBP by 3.4 mmHg (95% CI: −2.5 to −4.3 mmHg) and DBP by 1.5 mmHg (95% CI: −1.0 to −2.1 mmHg). The meta-analyses of three comparisons showed that SBP decreased by 3.5 mmHg (95% CI: −0.8 to −3.1 mmHg) and DBP by 1.8 mmHg (95% CI: −0.5 to −3.1 mmHg) when the achieved sodium intake of the intervention was less than 2 g/day as compared to at least 2 g/day. In the meta-analyses of 11 studies (2,339 participants) that reported changes in blood lipids, reduced sodium intake had no effects on total cholesterol (0.02 mmol/L; 95% CI: −0.03 to 0.07 mmol/l), LDL-cholesterol (0.03 mmol/l; 95% CI: −0.02 to 0.08 mmol/l), HDL-cholesterol (−0.01 mmol/L; 95% CI: −0.03 to 0.00 mmol/L) or triglycerides (0.04 mmol/L; 95% CI: −0.04 to 0.09 mmol/L). The four randomised trials reporting plasma adrenalin and seven trials reporting noradrenaline detected no effects of sodium restriction on plasma catecholamine levels.

The dose-response meta-analyses of Filippini et al. included 85 experimental studies with at least 4 weeks of follow-up, 24-h urinary sodium excretion measurements, dietary sodium changes by diet or supplementation, and measurements of SBP and DBP (55). The study reports were published between 1973 and 2018, and the analyses included over 10,000 individuals. The achieved difference in 24-h urinary sodium between the intervention and control groups ranged from 5 to 309 mmol/day (0.1–7.1 g/day) with a median difference of 80 mmol/day (1.8 g/day). According to linear regression analyses, every 100 mmol/day (2.3 g/day) reduction in urinary sodium excretion was associated with a lower SBP of 5.6 mmHg (95% CI: −6.6 to −4.5 mmHg) and a 2.3 mmHg lower DBP (95% CI: −3.3 to −1.7 mmHg). Correspondingly, every 1 g/day decrease in sodium excretion was associated with a lower SBP of 2.4 mmHg (95% CI: −2.9 to −2.0 mmHg) and a 1.0 mmHg lower DBP (95% CI: −1.3 to −0.7 mmHg). A dose-response relationship was observed with a mean SBP/DBP reduction of 6.5 mmHg (95% CI: −7.8 to −5.2 mmHg)/3.0 mmHg (95% CI: −3.7 to −2.3 mmHg) among hypertensives and a reduction of 2.3 mmHg (95% CI: −3.3 to −1.3 mmHg)/0.8 mmHg (95% CI: −1.9 to +0.3 mmHg) among normotensives per 100 mmol/day (2.3 g/day) reduction in sodium excretion. The approximately linear relationship between achieved sodium intake and mean SBP and DBP extended down to sodium excretion of 1.5 g/day. As compared to sodium excretion of 2.0 g/day, sodium excretion of 1.5 g/day was associated with a lower SBP of 1.3 mmHg (95% CI: −1.8 to −0.8 mmHg) and a lower DBP of 0.4 mmHg (95% CI: −0.8 to −0.0 mmHg). Correspondingly, an increase in sodium excretion from 2 to 2.5 g/day was associated with a higher SBP of 1.3 mmHg (95% CI: +0.6 to +1.7 mmHg) and a higher DBP of 0.4 mmHg (95% CI: +0.1 to +0.6 mmHg).

Only a few studies have examined the long-term effects on BP of sodium restriction. Jula et al. (56) studied the effects on BP, serum lipids and echocardiographically assessed heart structure and function of a non-pharmacological treatment based mainly on sodium restriction in a 12-month controlled randomised study with 91 middle-aged untreated and mildly hypertensive men and women. The estimated daily sodium intakes, calculated from 24 h urine samples, decreased in men from 227 mmol/day (5.2 g/day) to a mean level of 105 mmol/day (2.4 g/day), and in women from 129 mmol/day (3.0 g/day) to a mean level of 63 mmol/day (1.4 g/day). After 12 months non-pharmacological treatment, the mean weight in men was 1.9 kg lower and in women 0.3 kg higher compared to baseline. In the treatment group, energy derived from fat decreased in men by 4% and in women by 3% reflecting decreased intake of saturated and monounsaturated fats. The net BP decrease (the difference in changes between the treatment and control groups) during the 12 months was 8.2 mmHg for SBP and 5.8 mmHg for DBP in men and 9.5 mmHg for SBP and 5.6 mmHg for DBP in women. All changes were significant. In the treatment group, LDL-cholesterol decreased by 6.8% in men and by 12.1% in women. The non-pharmacological treatment decreased left ventricular mass (LVM) by 5.4%, and the decrease was larger (8.6%) in those subjects with baseline LVM/body surface area above the median values of both men and women (57). Regression of left ventricular hypertrophy was associated with decreased plasma levels of atrial natriuretic peptide (ANP), indicating decreased load of the heart (58). The study illustrated that a 1-year nonpharmacological treatment with reduced saturated fat intake partly compensated by increased unsaturated fat intake together with sodium restriction down to 2.4 g/day in men and down to 1.5 g/day in women, decreased statistically and clinically significantly both BP and LDL-cholesterol, and had favourable effects on the left ventricle of the heart.

In the Dietary Approaches to Stop Hypertension (DASH) trials, the effects of various controlled diets on the BP of adult Americans with normal or moderately elevated BP were studied (59, 60). The study assessed the influence of sodium intake on BP in 412 subjects and on fasting serum lipids in 390 subjects who were randomly assigned to follow either a control diet typical of the sodium intake in the US or to follow the DASH diet (60, 61). In both groups, a second randomisation was done, and the subjects followed their assigned diets at three sodium levels for 30 days in random order in a crossover design. The subjects were selected among adults 22 years or older who were not taking antihypertensive medication and who had a SBP ranging from 120 mmHg to 160 mmHg and a DBP ranging from 80 mmHg to 95 mmHg. The control diet had a fat composition corresponding to the usual American diet (36 E% total fat, 14 E% saturated fat), but a low content of fruits, vegetables, and dairy products. The DASH diet was rich in fruits, vegetables, and low-fat dairy products, but low in edible fats, snacks, and sweets, and had a low content of total fat (25 E%), saturated fat (7 E%), and cholesterol. The content of calcium, potassium, and magnesium in the control diet was lower than in the average US diet, but was higher in the DASH diet. The intake of dietary fibre was similar in both groups. Within the assigned diets, sodium levels were adjusted to provide a daily intake of 150 mmol (3.5 g, defined as high), 100 mmol (2.3 g, defined as intermediate), and 50 mmol (1.2 g, defined as low) for 30 consecutive days each, in random order. The average sodium intake, calculated from 24 h urine samples, was 141–144 mmol/day (3.3 g/day) during the high sodium phase, 64–67 mmol/day (1.5 g/day) during the low sodium phase and 106–107 mmol/day (2.4 g/day) during the intermediate sodium phase. Reduction of sodium intake from the high to the intermediate level significantly decreased SBP by 2.1 mmHg during the control diet and by 1.3 mmHg during the DASH diet. A further reduction from the intermediate to the low level caused additional reductions of 4.6 mmHg during the control diet and 1.7 mmHg during the DASH diet. A regression analysis of these data showed that a reduction in the sodium intake of 100 mmol (2.3 g) per day would decrease SBP by 3 mmHg in the DASH diet group and by 7 mmHg in the control diet group. Corresponding values for DBP were 1.5–2 mmHg and about 3 mmHg, respectively. The effects of sodium were observed in normotensive and hypertensive subjects, in subjects of different ethnicities, and in both women and men. The effects were not dependent on weight, but were higher in older (>45 years) than in younger subjects (59, 60). Total cholesterol was 0.41 mmol/l (7.8%) and LDL-cholesterol 0.33 mmol/l (9.7%) lower during the DASH diet than during the control diet (61). No differences were seen in fasting blood lipids across the three sodium intake phases (61).

The DASH trials clearly showed a BP lowering effect of sodium restriction, ranging from 1.5 to 2.4 g/day and finally to 3.5 g/day, that was independent of other dietary and lifestyle factors. An important finding was that the BP reduction was larger in the control group than in the DASH diet group. This implies that the benefits of sodium restriction are more pronounced among persons consuming a diet that is less than optimal in terms of fat, fruit, vegetable and low-fat dairy food intake (which is similar to the current dietary patterns in the Nordic countries) than among those already consuming a diet in line with the general nutrition recommendations (23). It should also be noted that the estimated daily potassium intakes, calculated from 24 h urine samples, indicated a potassium intake of 40–42 mmol/day (1.6 g/day) in the control group and 75–81 mmol/day (2.9–3.2 g/day) in the DASH diet group during the high, intermediate, and low sodium intake phases. It is also noteworthy that the mean daily dietary intake of potassium in the Nordic and Baltic countries is approximately twice as high as that of the DASH control group, and even somewhat higher than that of the DASH diet group (23). In the DASH trial, an interaction between dietary sodium and potassium may explain why the BP-lowering effect of sodium was higher during the control diet with a low potassium intake and lower, but still clinically significant, during the DASH diet with a high potassium intake. Sodium restriction from 3.3 to 2.4 g/day and down to 1.5 g/day had no effects on blood lipids. A limitation of the study was the relatively short duration (30 days) of the sodium restriction phases.

#### Children and adolescents

The BP of children living in industrialised countries rises with age, and the increase is more rapid in children of hypertensive parents than in children of normotensive parents (62–65). In the Finnish Special Turku Coronary Risk Factor Intervention Project (STRIP) study (65), SBP of children living in southwestern Finland increased with age along with sodium intake. At the age of 15 years, their SBP exceeded by 15–20 mmHg that of the adults in populations consuming extremely low-sodium diets (Brazil: Xingu and Yanomamo), and was 114 mmHg in girls and 121 mmHg in boys. The mean daily sodium intake was 1.5 g at the age of 13 months and 3.0 g at the age of 15 years. Similar levels of salt intake by children have been reported in other countries (66, 67). Childhood BP tracks with adult BP (68, 69) and predicts early signs of atherosclerosis in adolescence (70) and adulthood (70, 71).

A randomised trial among 476 Dutch newborn infants studied the effect of a low-sodium (average 120 mg/day) or a normal-sodium (average 330 mg/day) diet on BP during the first 6 months of life (72). The sodium intake in the low-sodium group was approximately the same as the intake of breastfed infants, whereas the intake in the normal-sodium group was similar to the sodium intake of infants fed commercial infant formula. At the end of the trial, SBP in the low-sodium group was 2.1 mmHg lower than in the control group. The authors also measured BP in 167 children from the original cohort (35% of the original study participants) after 15 years of follow-up. The adjusted SBP at follow-up was 3.6 mmHg lower and the DBP was 2.2 mmHg lower in adolescents who as infants had been assigned to the low-sodium group compared with those assigned to the control group.

Three meta-analyses of controlled trials have assessed effects of reduced salt intake on BP in children and adolescents (48, 54, 73). He and MacGregor identified 10 trials with 966 participants (73). The mean ages of the study participants ranged from 8 to 16 years, and the median duration of the trials was 4 weeks. A reduction in salt intake by 42% decreased SBP by 1.2 mmHg (95% CI: −0.6 to −1.8 mmHg) and DBP by 1.3 mmHg (95% CI: −0.7 to −1.9 mmHg). In the analyses of three trials with infants and with a median duration of 20 weeks, a reduction in sodium excretion by 54% decreased SBP by 2.5 mmHg (95% CI: −0.9 to −4.0 mmHg). Aburto et al. identified nine studies with 1,299 participants aged 2.6 to 19.8 years, and found that a reduction in sodium intake reduces SBP by 0.8 mmHg (95% CI: −0.3 to −1.4 mmHg) and DBP by 0.9 mmHg (95% CI: −0.1 to −1.2 mmHg) (54). Leyvraz et al. included 13 studies with 1,687 participants and with a mean duration of 16 weeks, and found that 1.2 g/day reduction in daily sodium intake decreased SBP by 0.6 mm Hg (95% CI: −0.5 to −0.8 mmHg) and DBP by 1.2 mmHg (95% CI: −0.4 to −1.9 mmHg) (48).

### Cardiovascular diseases

A number of prospective cohort studies, systematic reviews and meta-analyses have investigated the association between dietary salt intake and the risk of stroke and other cardiovascular events. Sodium intake has been estimated in the studies by dietary questionnaires (24-h dietary recalls, food diaries, FFQs), by measuring 24-h urinary sodium excretion or by spot urine samples and estimation equations. Twenty-four-hour urinary sodium is the gold standard method to estimate sodium intake. Because of a large daily variation in sodium excretion, multiple measurements over a longer time is needed to assess an individual’s usual longer term sodium intake (see above).

The comparative effectiveness review of AHRQ assessed the relation of sodium intake with CVD, CVD morbidity, CVD mortality, and total mortality (19). They concluded that sodium levels appear to be associated with risk of all-cause mortality, but the shape of the relationship could not be determined because of insufficient source of error. They concluded also that evidence was insufficient to assess association of sodium level and risk for CVD, coronary heart disease (CHD), or stroke morbidity and mortality. The assessment was based on 13 prospective cohort studies (15 cohorts) and one nested case-control study. Sodium intake was assessed by 24-h urinary sodium excretion in five cohorts, by overnight sodium excretion in one cohort, by spot urine samples in five cohorts, by 24-h dietary recall in three cohorts, and by 3-day food records in one cohort. It should be noted that the cohort studies using imprecise assessment methods of sodium intake carry a high risk of bias and are not suitable for assessments of CVD risk in relation to sodium intake. According to random-effects model meta-analyses of four studies with measured 24-h urinary excretion, the adjusted risks of total mortality increased (relative risk [RR]: 1.09; 95% CI: 1.00 to 1.19) per 50 mmol/l (1,150 mg/day) increase in urinary sodium excretion in generally healthy populations. One of the five studies that measured 24-h urinary sodium excretion found J-shape associations between sodium intake and mortality, and negative associations between higher sodium intake and CVD mortality. The prospective study was based on two cohorts, the Flemish Study on Genes, Environment and Health Outcomes (FLEMENGHO) and the European Project on Genes in Hypertension (EPOGH) (74). The outcome cohort consisted of 3,681 participants (2,674 in the FLEMENGHO and 1,007 in the EPOGH cohort). During the median follow-up time of 7.9 years, 84 fatal (81 in the FLEMENGHO and three in the EPOGH cohort) and 134 nonfatal outcomes (113 in the FLEMENGHO and 21 in the EPOGH cohort) were registered. Pulmonary embolism (*n* = 11) and pulmonary heart disease (*n* = 14) were unorthodoxly included as cardiovascular outcomes. The number of fatal (*n* = 29) and nonfatal (*n* = 43) heart failures was high, as 52 participants with CVD at baseline were excluded from the outcome cohort. Men and women in the lowest 24-h urinary sodium excretion tertile were slightly older and had lower body weight than men and women in the medium and high tertiles. Lower urinary volume and creatinine excretion suggest misclassification of participants and/or selection of more fragile subjects with lower muscle mass into the lowest sodium excretion tertile. The risk of bias in the FLEMENGHO and EPOGH cohort was unexpectedly estimated as medium instead of high by the AHRQ.

A systematic review and meta-analysis of prospective studies published between 1996 and 2008 assessed the relation between habitual salt intake and stroke or total cardiovascular events (75). The analysis included 19 independent cohorts from 13 studies with a total of 177,025 participants from the US, Finland, Japan, the Netherlands, Scotland and Taiwan, and over 11,000 vascular events. Follow-up varied between 3.5 and 19.0 years. Sodium intake was assessed by 24-h dietary recall (*n* = 4), FFQ (*n* = 3), FFQ and overnight urinary sodium excretion (*n* = 1), 24-h urinary sodium excretion (*n* = 4), or by a questionnaire (*n* = 1). A higher estimated sodium intake of 100 mmol/day (2.3 g/day) was associated with a higher incidence of stroke (pooled relative risk 1.23; 95% CI: 1.06 to 1.43) and CVD (RR: 1.14; 95% CI: 0.99 to 1.32). Higher sodium intake was associated significantly with higher CVD risk (RR: 1.17, 95% CI: 1.02 to 1.34) when one study with 24-h urine samples collected 5 days after the subjects had been asked to avoid consumption of foods with a high salt content was not included in the analyses (76).

The systematic review and meta-analyses of Aburto et al. included 12 prospective cohort studies (54). Sodium intake was assessed by 24-h dietary recall (*n* = 3), by FFQ (*n* = 3), 24-h urinary sodium excretion (*n* = 4), overnight urinary sodium excretion (*n* = 1), or was estimated from spot urine samples with Kawasaki equation (*n* = 1). The meta-analyses of three studies and five comparisons find an increased risk of CHD mortality with higher sodium intake (RR: 1.32, 95% CI: 1.13 to 1.53) (41). Meta-analyses of 10 studies detected an increased risk of stroke (RR: 1.24, 95% CI: 1.08 to 1.43) and an increased risk of fatal stroke (RR: 1.63, 95% CI: 1.27 to 2.10) along with higher sodium intake. The meta-analyses of sodium intake and CVD and CHD were inconclusive.

Poggio et al. performed a systematic review and meta-analyses of 11 studies and 229,785 participants representing the general population (77). The average follow-up time was 13.4 years (range 5.5 to 19.0 years). Sodium intake was assessed by 24-h dietary recall (*n* = 3), FFQ (*n* = 3), FFQ and 3-day dietary record (*n* = 1), or by 24-h urinary sodium excretion (*n* = 5). Higher sodium intake was associated with higher cardiovascular mortality (RR: 1.12; 95% CI: 1.06 to 1.19). The meta-regression analyses showed that CVD mortality increased by 1% (*P* = 0.016) for every increase of 10 mmol/day (230 mg/day) in sodium intake.

Wang et al. assessed the relation between sodium intake and CVD with a systematic review and meta-analysis of 36 studies, 616,905 participants, and a follow-up time ranging from 2.7 to 29 years (78). Sodium intake was assessed by 24-h dietary recall (*n* = 8), FFQ (*n* = 7), dietary records (*n* = 2), self-administered questionnaire (*n* = 1), 24-h urinary sodium excretion (*n* = 18), or by spot urine sodium excretion (*n* = 1). Higher sodium intake was associated with higher adjusted risk of CVD (RR: 1.19; 95% CI: 1.08 to 1.30). According to dose-response analyses of 20 studies, the risk of CVD increased linearly up to 6% for every 1 g increase in dietary sodium intake.

Ma et al. assessed relations of sodium intake and cardiovascular risk by including individual-participant data from six prospective cohorts of generally healthy subjects (79). Sodium intake was assessed with the use of at least two 24-h urine samples per participant in six different cohorts including 10,709 participants with a mean age of 51.5 years. During the median follow-up time of 8.8 years, 571 cardiovascular events (coronary revascularisation, fatal or nonfatal myocardial infarction, fatal or nonfatal stroke) were ascertained. Higher sodium excretion, lower potassium excretion and higher sodium/potassium ratio were associated with a higher cardiovascular event risk in analyses adjusted for all confounding factors. In analyses comparing the highest sodium excretion quartile (median 4.7 g/day) with the lowest quartile (median 2.2 g/day), higher sodium excretion was associated with an increased risk of cardiovascular event (hazard ratio [HR] 1.60; 95% CI: 1.19 to 2.14). The spline analyses of the data showed a linear association over the range of sodium excretion. According to dose-response analyses, the risk of a cardiovascular event increased by 18% for every 1 g increase in daily sodium excretion (HR: 1.18; 95% CI: 1.08 to 1.29).

Prospective studies based on a single daily sodium intake estimate from spot urine of the participants have shown J-shaped association between estimated sodium intake and CVD (15, 16). Sodium intake estimates in these studies have been made from morning spot urine samples by using the Kawasaki formula, which at the individual level underestimates high and overestimates low sodium intake and at the study population level overestimates average sodium intake (17).

The observational analyses of two large randomised drug-treatment studies, ONTARGET (Ongoing Telmisartan Alone and in combination with Ramipril Global Endpoint Trial) and TRANSCEND (Telmisartan Randomized Assessment Study in ACE Intolerant Subjects with CVD), included two cohorts with 28,880 patients from 733 centres in 40 countries (15). Both trials included patients with a high risk of CVD (age ≥ 55 years with established CVD or high-risk diabetes). According to medical history, 48% of the study population had suffered from a previous myocardial infarction, 21% from a stroke, 70% had hypertension, and 37% had diabetes. The mean follow-up time was 56 months. The main outcome measures were cardiovascular death, myocardial infarction, stroke and hospitalisation for congestive heart failure. Subjects were divided into seven groups according to their estimated baseline sodium intake (<2 g/day (*n* = 818), 2.00–2.99 g/day (*n* = 2,654), 3.00–3.99 g/day (*n* = 5,699), 4.00–5.99 g/day (*n* = 14,156), 6.00–6.99 g/day (*n* = 3,380), 7.00–8.00 g/day (*n* = 1,326), and >8.00 g/day (*n* = 847). The use of diuretics, the history of hypertension and diabetes, and the prevalence of atrial fibrillation was highest among the groups with an estimated sodium intake of <2.0 and >8.0 g/day and lowest in the reference group with an estimated sodium intake of 4.00–5.99 g/day. Compared with a baseline sodium excretion of 4.00 to 5.99 g/day, sodium excretion greater than 7.0 g/day was associated with an increased risk of myocardial infarction, stroke, cardiovascular death, and congestive heart failure. Sodium excretion of less than 3.0 g/day was associated with an increased risk of congestive heart failure and cardiovascular death, but not with an increased risk of myocardial infarction or stroke. Reverse causality and inaccurate estimates of the participant’s sodium intake are the plausible explanations for the observed J-shape associations between sodium intake, congestive heart failure and cardiovascular death.

The Prospective Urban Rural Epidemiology (PURE) study enrolled and followed 156,424 subjects, aged 35 to 70 years, living in 628 urban and rural communities in 17 countries (16). A morning fasting urine sample was obtained and daily sodium intake was estimated by using the Kawasaki formula. The mean follow-up time was 3.7 years. The primary outcome was defined as death from any cause or a cardiovascular event (cardiovascular death, stroke, myocardial infarction or heart failure). The study included 101,945 participants of whom 42% were from China. The mean estimated daily sodium intake was 4.9 g. A primary outcome occurred in 3,317 participants. As compared with the reference group with an estimated sodium intake of 4.00–5.99 g/day, a sodium intake of ≥7.0 g/day was associated with increased risk of the primary outcome and of its components. Respectively, an estimated sodium intake of <3.0 g/day was associated with an increased risk of the primary outcome. As compared with the reference group, subjects in the low sodium group were older, had a higher proportion of women, subjects using cardiovascular medication or having a history of CVD. Reverse causality and selection bias into different salt groups by the inaccurate estimation method of individual sodium intake are the plausible explanators of the U-shaped association of sodium intake and cardiovascular events in the PURE study.

The effects of predictive formulas on the estimation of sodium intake and the associations of these estimates with mortality was analysed using TOHP follow-up data (17). The analyses included 2,974 individuals aged 30 to 54 years who were not assigned to sodium intervention. During the follow-up of 24 years, 272 deaths occurred. Sodium intake was assessed from three to seven 24-h urinary sodium collections by direct measurements of 24-h sodium excretion and by three sodium estimation formulas. As compared to measured average 24-h urinary sodium of 3.8 g/day over the trial, the estimated average 24-h urinary sodium based on the Kawasaki formula overestimated sodium intake by 1.3 g/day. There was a significant linear relationship between the average measured daily sodium excretion and all-cause mortality. The relationships between average sodium intake and mortality were J- or U-shaped when sodium intake was estimated by using the spot-urine-based formulas of Kawasaki, Tanaka or Intersalt. The study shows clearly that even average sodium intake estimated from urine samples over a long time by using spot-urine-based formulas is not a valid method to assess health risks in association with sodium intake. The study confirms the findings of other studies (79, 80) that 24-h urinary sodium averaged over several measurements and longer time is the gold standard in the estimation of an individual’s usual sodium intake and the health risks associated with sodium intake.

#### Intervention studies

A recent cluster-RCT involving 20,995 persons from 600 villages in rural China studied the effect of potassium enriched salt substitute on CVD (81). The mean age of the participants was 65.4 years, 73% of the participants had a history of stroke, and 88% had a history of hypertension. Twenty-four-hour urine samples were collected from a representative sample of study subjects at baseline and at 12-month intervals during the study. During the mean follow-up time of 4.7 years, 4,172 deaths occurred. The baseline sodium and potassium intakes were 4.3 and 1.4 g/day, respectively. During the follow-up the, the average sodium intake was 0.35 g/day lower, the potassium intake 0.80 g/day higher, and the average SBP 3.3 mmHg lower in the salt substitute group. Compared to controls, significantly less strokes (RR: 0.86; 95% CI: 0.77 to 0.96), major cardiovascular events (RR: 0.86; 95% CI: 0.80 to 0.94), and deaths from any causes (RR: 0.88; 95% CI: 0.82 to 0.95) occurred in the salt substitute group.

Population follow-up data (‘natural non-randomised interventions’) have also shown that BP and CVD mortality decreases along with decreased sodium intake and other favourable dietary changes. In Finland, salt intake decreased by 40% from the 1970s to 2002 along with a decreased intake of saturated fats and increased intake of fruits and vegetables (82–84). The dietary changes were associated with a 10–20 mmHg and 6–10 mmHg decrease in population SBP and DBP, respectively, and with a 70% decrease in stroke and CHD mortality (85, 86). The observed decrease in BP was much larger than expected from cross-sectional and trial data. This suggest that the effect of dietary sodium on the vasculature and BP rise accumulates with age, and that the current estimates underreport the effects on BP of a long-term population-based prevention with decreased sodium intake.

### Other health outcomes

Sodium reduction decreases albuminuria and proteinuria, markers of kidney glomerular damage. The meta-analysis of Nomura et al. included 20 studies with 766 participants and a mean study duration of 14 days (range 4 to186 days) (87). The renal outcomes (albuminuria, albumin-to-creatinine ratio, proteinuria, protein-to creatinine ratio) differed between the studies. The pooled effects size showed a 53% (95% CI: 21 to 84%) higher albumin or protein excretion on the high-sodium than on the low-sodium diet. The meta-analysis of D’Elia et al. included 11 studies, 23 cohorts and 516 participants with a follow-up time of 1–6 weeks (88). An average reduction in sodium intake of 92 mmol/day (2.1 g/day) was associated with a 32% (95% CI: −44 to −19%) reduction in urinary albumin excretion. The effect was higher in cohorts using renin-angiotensin-aldosterone-blocking medication, in studies with a duration of at least 2 weeks, and in patients with evidence of kidney damage. Sufficiently powered long-term intervention studies are needed to assess if sodium restriction can prevent glomerular damages in high-risk populations, and, slow down worsening of kidney function and development of end stage renal disease in subjects with kidney disease.

Several studies have indicated a positive relationship between sodium excretion and calcium excretion and that sodium intake might play a role in the aetiology of osteoporosis and kidney stones (89, 90).

Two prospective cohort studies have assessed the risk between sodium intake and diabetes (91, 92). The first study included 1,935 men and women, aged 35–64 years from the WHO FINMONICA 1982 and 1987 cohorts who had complete data on 24-h urinary sodium (91). During the mean follow-up time of 18.1 years, 129 incident cases of type 2 diabetes occurred. The multivariate adjusted HR for diabetes in the highest (>6.0 g/day in men and >4.5 g/day in women) versus lowest quartile (<3.7 g in men and <2.8 g/day in women) of 24-h urinary sodium was 2.05 (95% CI: 1.43 to 2.96). In the second study, 4,630 men and women aged 25–64 years collected 24-h urine samples between 1979 and 2002, and were followed up to 14 years (92). The study subjects of the population-based samples comprised of participants of the North-Karelia Salt Project in 1979, WHO FINMONICA studies 1982 and 1987, and the National FINRISK Study in 2002. During the follow-up, 161 new cases of diabetes were observed. Compared with the highest quartile of sodium intake (mean 6.7 g/day) persons in the lowest quartile (mean 2.2 g/day) had a lower multivariate adjusted HR of diabetes (HR: 0.52; 95% CI: 0.32–0.87) (92). A systematic review and meta- analyses of 20 randomised studies, 504 healthy hypertensive participants aged 10 to 79 years and with a mean duration of approximately 11 days (range 3 to 56 days) assessed effects of sodium restriction on fasting serum glucose (93). A sodium restriction from a mean of 4.8 to a mean of 1.1 g/day did not change fasting serum glucose. The difference in glucose from low to high sodium intake was 0.01 mmol/l (95% CI: −0.04 to 0.05 mmol/l). Sufficiently powered long-term sodium restriction trials are needed to assess whether low sodium intake can prevent glucose intolerance and type 2 diabetes.

The World Cancer Research Fund/American Institute for Cancer Research (WCRF/AICR) concluded that there is probable evidence that total intake of salt and sodium is associated with stomach cancer (94). A meta-analysis of seven prospective cohort studies found an increased risk with dietary intakes categorised as ‘high’ or ‘moderately high’ compared to ‘low’ intakes (95). The meta-analysis included 10 cohorts, 268,718 participants, and 1,474 events with a follow-up of at least 4 years. The categorisation of intakes was based on reported tertiles or middle and extreme quintiles. Two newer systematic review and meta-analyses replicated the finding of a positive association between salt intake and gastric cancer (96, 97). High salt intake can change the viscosity of mucus, increase gastrin secretion and inflammatory responses, enhance epithelial damage and epithelial cell proliferation, and it may have synergistic effects with Helicobacter pylori infection (98). Damages in the gastric mucosa may make it also vulnerable to carcinogenic compounds in the food.

High salt intake may play a role in the development of autoimmune disease. The studies with autoimmune animal models have shown that a high sodium diet can induce the development of multiple sclerosis (MS), inflammatory bowel disease, rheumatoid arthritis, and lupus nephritis. High salt intake has been suggested to drive lymphocyte differentiation from regulatory T cells towards proinflammatory T-helper (Th)-1 and Th17 phenotypes, and to lead in changes in the gut microbiota (99, 100). Data from human studies of the relations between sodium intake and autoimmune diseases are sparse. One large cross-sectional study from Spain, based on health questionnaires, and several case-control studies suggest that a high salt intake is associated with a higher risk of rheumatoid arthritis (101). The association of salt and MS has been assessed in three studies as the time of remission to relapse of the disease or as the time from the onset of clinically isolated symptoms (CIS) to the onset of multiple signs of the disease (99). One study showed an inverse association between sodium intake and remission time, but the two other studies did not observe any significant associations between sodium intake and remission time or conversion time from CIS to MS.

## Requirement and recommended intakes

### Adults

Among adults, sodium balance can be maintained at intakes as low as 10 mmol (230 mg) per day, which corresponds to about 0.6 g of salt (102, 103). An intake of 25 mmol (575 mg) per day, corresponding to about 1.5 g salt, is set as the estimated lower intake level and accounts for variations in physical activity and climate (102). This is line with findings from populations using no added salt in their diet.

The average BP is approximately 100/60 mmHg and does not rise with age in populations with a very low sodium intake (<0.8 g/day), and the rise with age is only gently sloping in populations with a sodium intake between 0.8 and 1.3 g/day (44). A positive linear correlation between sodium intake and BP has been confirmed in cross-sectional and observational studies using proper measurement techniques of sodium intake. According to a recent meta-analysis, there is a dose-relationship between sodium reduction and BP decrease and a significant BP decrease from an intake of 2.5 g/day to an intake of 2 g/day (55). Several intervention studies have confirmed the efficiency and safety of reducing blood sodium intake to a level of less than 2 g/day. Analyses assessing sodium intake using at least two 24-h urine samples have shown a linear positive relationship between sodium intake and the risk of a cardiovascular event or death. The optimal sodium intake goal would be probably about 1.5 g/day.

Based on available data, a limitation of sodium intake to 2 g/day – corresponding to 5 g salt (NaCl) – is suggested.

### Children

BP rises along with increasing sodium *intake* from early childhood to late adulthood. SBP may already at the age of 15 years exceed by 15–20 mmHg that of adults in populations consuming very low-sodium diets. The BP rise with age is a consequence of structural and functional changes in the vasculature. BP measured in childhood tracks with the level measured in adulthood and predicts early atherosclerosis in adulthood. The available data suggest that a reduction in sodium intake at a young age is associated with lower BP in later life. Following a lifelong salt-reduced diet beginning in early childhood is recommended. It is also prudent to limit sodium intake in childhood in order to avoid preference for a diet with a high salt content.

### Pregnancy and lactation

Pregnancy and lactation are associated with a small increase in the physiological requirements for sodium by about 0.07 g or 3 mmol per day (pregnancy) and 0.13 g or 6 mmol per day (full lactation) (4). These amounts are small and can apparently be handled by the body’s homeostatic system. There is a lack of evidence to suggest that sodium requirements during pregnancy and lactation differ significantly from that of non-pregnant women.

### International expert reports

The sodium intake recommendation of NNR2023 is in line with the recommendations of WHO (104, 105), EFSA (4) and the National Health and Medical Research Council (NHMRC) of Australia and New Zealand (106). The EFSA Panel considers that 2.0 g sodium/day is safe and adequate intake (AI) for the general European Union population of adults (4). The British Expert Panel have published recommendations to limit salt intake to 6 g/day among adults (107). The expert report of the nutrition societies of Germany, Austria, and Switzerland set a reference value of 1.5 g/day for sodium intake in adults (108). The American consensus study report of the National Academies of Sciences, Engineering and Medicine set also the reference level (AI) of sodium intake of adults to 1.5 g/day, and suggested to reduce the intake if above 2.3 g/day (19). The American Heart Association (AHA) and the American College of Cardiology (ACC) suggest to reduce sodium intake to less than 1.5 g/day in the prevention and treatment of hypertension (109, 110). The importance of population-wide sodium reduction as a means to prevent CVD and stroke has been pointed out by WHO, the AHA (111), and the British National Institute for Health and Clinical Excellence (112).
